# *In vivo* photoacoustic monitoring using 700-nm region Raman source for targeting Prussian blue nanoparticles in mouse tumor model

**DOI:** 10.1038/s41598-018-20139-0

**Published:** 2018-01-31

**Authors:** Nhat Quang Bui, Soon-Woo Cho, Madhappan Santha Moorthy, Sang Min Park, Zhonglie Piao, Seung Yun Nam, Hyun Wook Kang, Chang-Seok Kim, Junghwan Oh

**Affiliations:** 1 Interdisciplinary Program of Biomedical Mechanical & Electrical Engineering, Pukyong National University, Busan, 48513 Republic of Korea; 2 Department of Cogno-Mechatronics Engineering, Pusan National University, Busan, 46241 Republic of Korea; 3 Center for Marine-Integrated Biomedical Technology, Pukyong National University, Busan, 48513 Republic of Korea; 4 Department of Biomedical Engineering, Pukyong National University, Busan, 48513 Republic of Korea; 5 Massachusetts General Hospital, Wellman Center for Photomedicine, Boston, 02114 USA

## Abstract

Photoacoustic imaging (PAI) is a noninvasive imaging tool to visualize optical absorbing contrast agents. Due to high ultrasonic resolution and superior optical sensitivity, PAI can be used to monitor nanoparticle-mediated cancer therapy. The current study synthesized Food and Drug Administration-approved Prussian blue (PB) in the form of nanoparticles (NPs) with the peak absorption at 712 nm for photoacoustically imaging tumor-bearing mouse models. To monitor PB NPs from the background tissue *in vivo*, we also developed a new 700-nm-region stimulated Raman scattering (SRS) source (pulse energy up to 200 nJ and repetition rate up to 50 kHz) and implemented optical-resolution photoacoustic microscopy (OR-PAM). The SRS-assisted OR-PAM system was able to monitor PB NPs in the tumor model with micrometer resolution. Due to strong light absorption at 712 nm, the developed SRS light yielded a two-fold higher contrast from PB NPs, in comparison with a 532-nm pumping source. The proposed laser source involved cost-effective and simple system implementation along with high compatibility with the fiber-based OR-PAM system. The study highlights the OR-PAM system in conjunction with the tunable-color SRS light source as a feasible tool to assist NP-mediated cancer therapy.

## Introduction

Cancer has been a leading cause of world-wide human fatalities for many years^1^. There are various state-of-the-art cancer therapeutics including nanoparticle (NP)-mediated photothermal therapy (PTT), which is considered a promising cancer treatment method because it is noninvasive and cancer-specific^2–5^. Photoacoustic imaging (PAI) technique is utilized for both cancer detection and PTT guidance^2–11^. Specifically, PAI combines the advantages of high ultrasonic resolution and strong optical contrast and has attracted wide attention as an exciting noninvasive imaging modality for visualizing structural and functional information in biological tissues. For example, PAI is used for structural and functional imaging of brain, breast cancer imaging, and tumor angiogenesis monitoring^9,10,12^. Following a cancer diagnosis, PAI is used to monitor NP deposition at the tumor before PTT^2–8,10^. Additionally, PAI employs a nonionizing short-pulse light source to acoustically visualize biological tissues based on the optoacoustic effects that result from the formation of ultrasonic waves induced by the thermal expansion of optical absorbers in the tissue^9,12–15^. Furthermore, PAI exploits both unscattered and scattered light to image deeper tissues with high optical resolution compared to pure optical imaging modalities (e.g., optical coherence tomography or confocal microscopy). Moreover, high photoacoustic (PA) signal-to-noise ratio (SNR) is achieved by specifically selecting the excitation laser wavelength in the near-infrared (NIR) region to minimize the light scattering effect^16,17^. The strong PA signal from hemoglobin is known to be a major hindrance for imaging biological tissues. Therefore, several PAI applications use contrast agents to preferentially absorb light at NIR wavelengths, far from 532 nm, which is strongly absorbed by blood.

For several decades, numerous nanoscale NIR absorbers, including gold nanostructures^3,4,6,7,18–20^, carbon-based nanomaterials^21,22^, copper sulfide NPs^23^, and polypyrrole NPs^24^, have been widely explored as potential contrast agents for both PAI and PTT. Despite the encouraging results, all these absorbers are nonbiodegradable and typically remain in the body for long periods of time, which is eventually associated with high toxicity. Prussian blue (PB) is a dye known from ancient times that corresponds to a dark blue micro-crystalline pigment and is used as an antidote for certain types of heavy-metal poisoning in medicine^25,26^. It is important to note that PB NPs have been explored as an excellent contrast agent for both PAI and PTT and provide an alternative to traditional agents due to their superior absorption efficiency, good photothermal efficiency, and high photothermal stability under NIR laser irradiation. Additionally, PB NPs are fabricated from low-cost chemical agents and easy to synthesize. Importantly, PB is clinically approved by the Food and Drug Administration (FDA) owing to its outstanding biosafety for the human body^2,3,11,25^. However, the use of PB NPs for PAI is rarely reported as it may possibly be related to the lack of low-cost, high-repetition-rate, and suitable specific-wavelength light sources for targeting PB NPs.

For functional and spectroscopic PAI that requires multiple wavelengths, optical parametric oscillators and dye lasers are conventionally used as light sources. However, these laser systems are quite expensive and suffer from low repetition rates (under 1 kHz)^16,27^. With respect to high repetition rates, fiber microchip lasers were reported with a repetition rate exceeding 100 kHz; however, the operating wavelengths are typically fixed at 532 nm or 1064 nm^28,29^. Additionally, a high-repetition-rate (up to 10 kHz) pulsed laser diode was recently used for a low-cost PAI system based on a long laser pulse induced dual PA nonlinear effect^30^. Although it may be the cheapest solution for a low-cost PAI system, the laser diode restrains the spatial resolution of the PAI system to the millimeter level due to intrinsic physical constraints such as short thermal relaxation times, safety limits, and low sensitivity. In order to overcome the limitations of these light sources, a tunable-color fiber laser based on the stimulated Raman scattering (SRS) effect was reported^31–34^. To date, functional and spectroscopic PAI in conjunction with SRS sources has been demonstrated in the visible region at 532 nm, 546 nm, 560 nm, 580 nm, and 600 nm for phantom imaging^31–34^ and at 532 nm, 545 nm, and 558 nm for *in vivo* blood oxygenation^33,34^. However, there have been no studies exploiting the potential of an SRS laser source for PA monitoring of NPs with a specifically designed absorption peak wavelength. Moreover, PAI has been applied for tumor detection, where the PA image contrast is enhanced owing to the presence of dense vasculatures at the tumor sites compared to normal tissues^16,35^. The best optical wavelengths for visualizing superficial vasculatures are in the green region, while red and far-red wavelengths are optimal for deep tissue imaging and highly absorbed by PB NPs. Most importantly, the proposed SRS light source provides a potential excitation range from the green to red wavelengths and therefore can visualize PB NPs and blood vessels from in the background tissues.

In this study, we explored the feasibility of *in vivo* monitoring of PB NPs in a tumor-bearing mouse model by using a tunable-color optical-resolution photoacoustic microscopy (OR-PAM) system based on an SRS light source. The key features of the SRS light source are (1) the high repetition rate of up to 50 kHz, (2) wide range of wavelengths extending from 532 nm to 712 nm, (3) high pulse energies up to hundreds of nJ, (4) simple system implementation, (5) low cost, and (6) high compatibility with fiber-based OR-PAM systems owning to the use of a polarization-maintaining single-mode fiber (PM-SMF). In the experiment, PB NPs were synthesized and injected into a tumor-bearing mouse model. Finally, we successfully imaged the PB NPs decomposition in the mouse tumor following the intratumoral injection.

## Results

### Tunable-color OR-PAM

**Figure 1:**
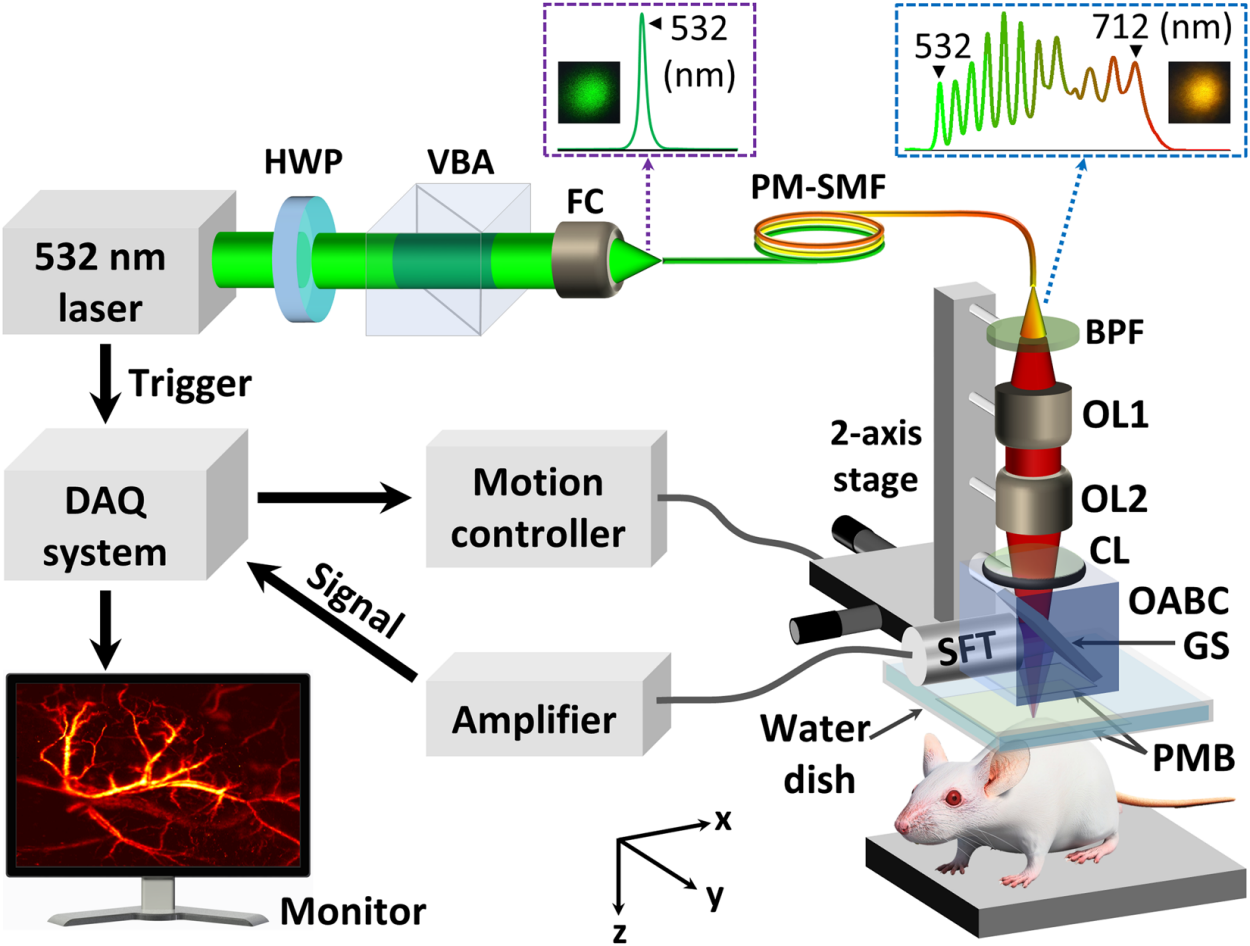
Schematic of tunable-color OR-PAM system. The purple and the blue dashed lines indicate the laser spectra, as well as the real light colors before and after PM-SMF optical fiber, respectively. HWP: half-wave plate; VBA: variable beam splitter/attenuator; FC: fiber coupler; PM-SMF: polarization-maintaining single-mode fiber; BPF: bandpass filter; OL: objective lens; CL: corrective lens; GS: glass slab; PMB: plastic membrane; SFT: spherically focused transducer; OABC: optical-acoustic beam combiner; DAQ: data acquisition.

**Figure 2:**
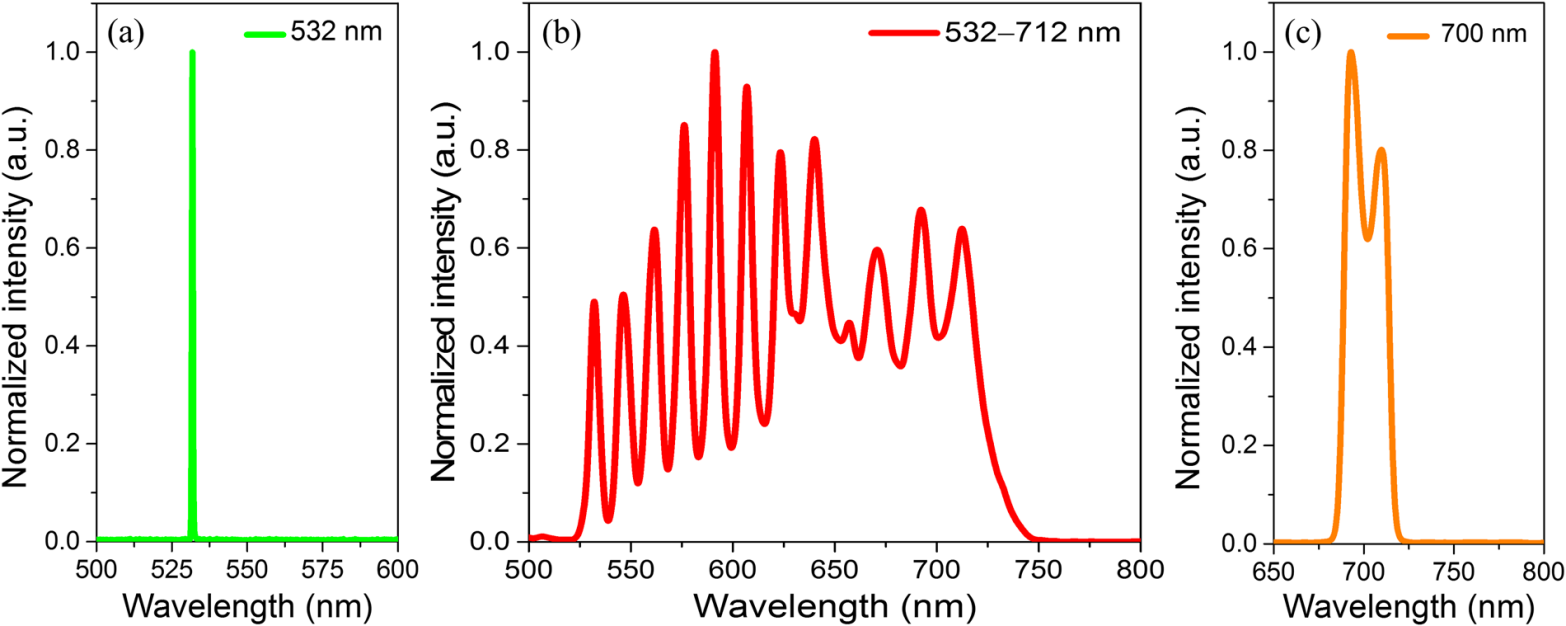
(**a**) Measured pumping light source spectra at PM-SMF input. Measured SRS light source spectra at PM-SMF output (**b**) without bandpass filter and (**c**) with 700 nm bandpass filter (FWHM of 25 nm).

Figure 1 shows a schematic diagram of the proposed tunable-color OR-PAM system. The detailed implementation of the system is described in the methods section. Briefly, the tunable-color OR-PAM system uses a PM-SMF deliver light from a compact 532-nm laser source. The light in the output of the PM-SMF is used to excite a sample to generate PA waves and is aligned confocally with a spherical focused transducer through a custom-made optical–acoustic beam combiner (OABC) to maximize the detection sensitivity. A two-axis linear stage provides volumetric imaging of the sample by raster-scanning the OABC along the x–y plane. The generated PA signals are then amplified and converted to PA images via Hilbert transformation. Figure 2(a) shows the pumping light source spectra at the input of the PM-SMF. The full power of the SRS light source outputs, after the PM-SMF, corresponds to an input pulse energy of 16 µJ, as shown in Fig. 2(b). The spectral properties of the optical fiber output were measured with a CCD-equipped spectrometer (CCS175/M, Thorlabs). Multiple discrete wavelengths exist in the output spectrum in a range from ~532 nm to 712 nm. We note that the shifted wavelength values are in accordance with the cascaded SRS effect of silica corresponding to ~13.2 THz for the Raman gain coefficient peak^36^. The pulse energy for the full spectrum was measured as 1.5 µJ using a broadband power meter (3A-SH-ROHS, Ophir Laser Measurement Group).

### Characterization of the PB NPs

**Figure 3:**
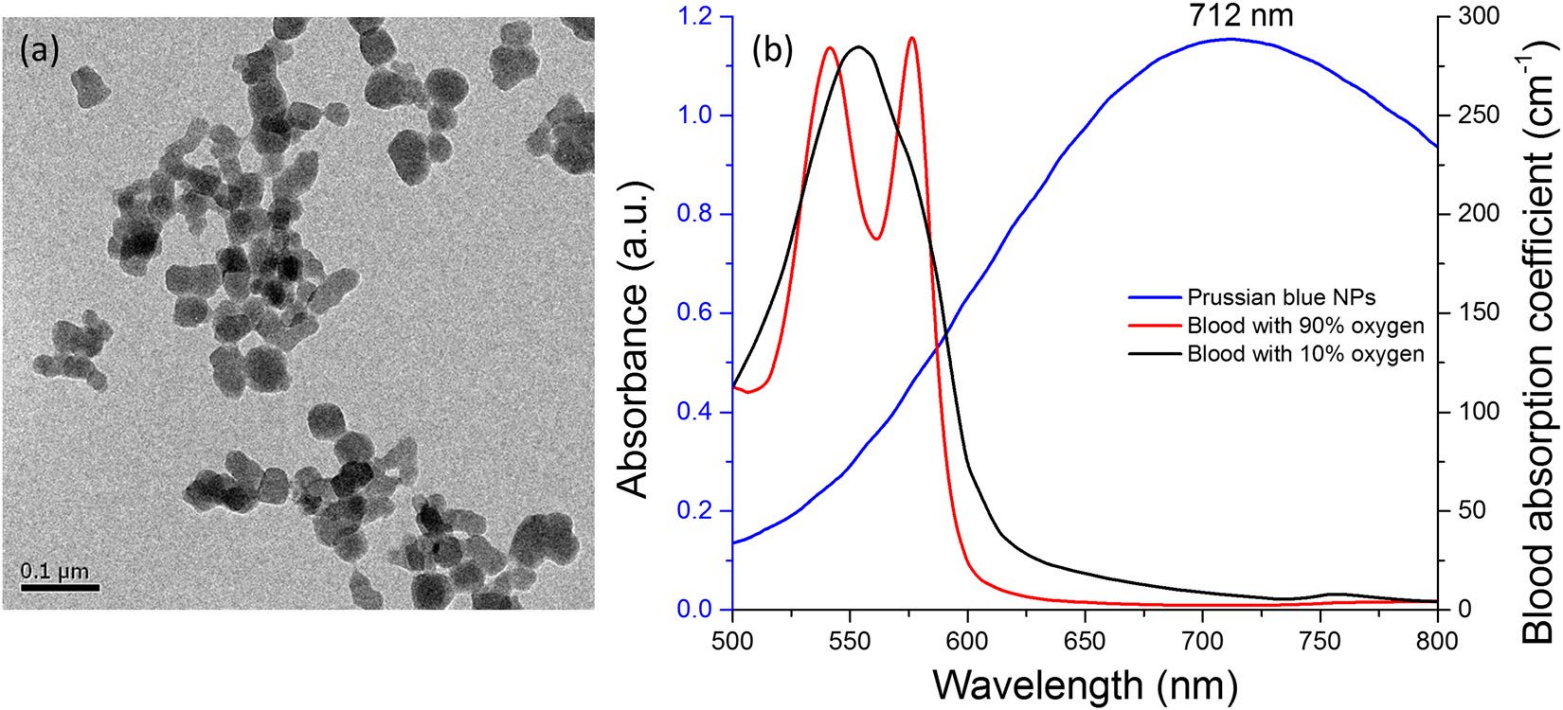
(**a**) TEM image of fabricated PB NPs and (**b**) measured absorption spectra of PB NPs (blue) and optical absorption coefficients of blood with 90% (red) and 10% (black) oxygen saturation adapted from Prahl^37^ and Kollias *et al*.^38^.

Figure 3(a) shows a transmission electron microscopy (TEM) image of the fabricated PB NPs. The average size of the NPs is 42 nm. The study examined measured absorption spectra of the PB NPs at a concentration of 0.5 mg/mL and optical absorption coefficients of blood with 90% as well as 10% oxygen saturation at normal concentrations in biological tissue as shown in Fig. 3(b) (150 g of hemoglobin and deoxyhemoglobin in 1 L of blood, data adapted from Prahl^37^ and Kollias *et al*.^38^). Typically, PB NPs exhibit a broad absorption band from 500 nm to 800 nm, with a strong absorption peak in the far-red region at 712 nm that corresponds to the energy of the metal–metal charge transfer between Fe(III) and Fe(II) via a cyanide bridge^39^. The absorption spectra suggest that the laser wavelength in the red region (i.e., 622–780 nm^40^) is close to the maximum absorption peak of the PB NPs, in contrast to the output wavelength of 532 nm. For arterial blood with 90% oxygen saturation, the optical absorption coefficients at 532 nm and 712 nm correspond to ~235 cm^−1^ and ~2.36 cm^−1^, respectively. Therefore, the 712 nm light is absorbed hundred times less efficiently than the 532 nm light; this implies better coupling of optical energy to PB NPs (i.e., minimal loss due to blood absorption).

### ***In vivo*** PA imaging

**Figure 4:**
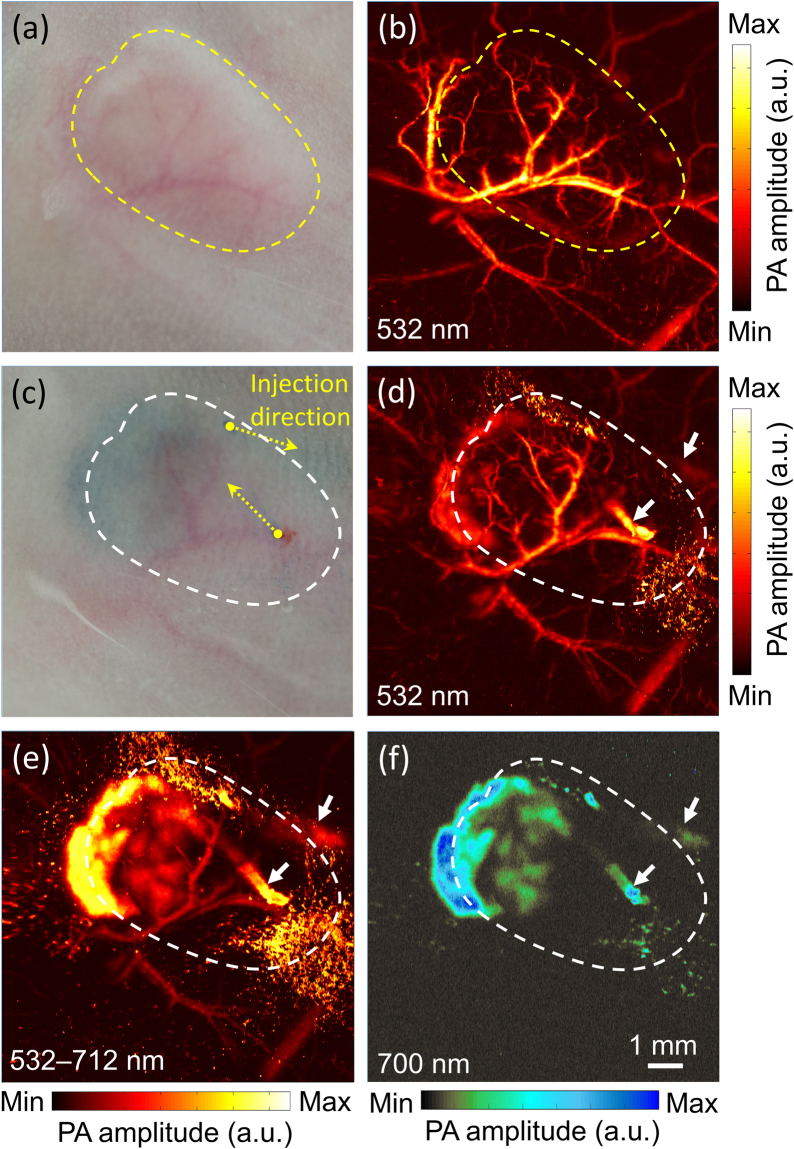
(**a**) and (**b**): Photograph and corresponding PA MAP image of blood vessels in mouse tumor model. (**c**–**f**): Photograph and corresponding PA MAP images of mouse tumor after injection of PB NPs. The dash lines indicate the tumor boundaries. The white arrows in (**d**–**f**) show the injected positions. The wavelength and the corresponding pulse energy were: (**b**) and (**d**) 532 nm, 0.8 µJ; (**e**) 532–712 nm (full spectrum), 1.5 µJ; (**f**) 700 ± 12.5 nm, 0.2 µJ.

In order to monitor PB NPs for NP-mediated PTT, a mouse tumor was photoacoustically imaged before and after the injection of PB NPs with 532 nm and tunable-color light. The PA image for the excitation wavelength of 532 nm was obtained with a 2-m single-mode fiber (SMF, P1-460B-FC-2, Thorlabs) and used as a reference for comparison with the results for tunable-color light. In ~20 min, the imaging system acquired a volumetric PA image over a 10 × 10 mm^2^ area in the x–y plane with a scanning step sizes of 2 µm and 20 µm in the x and y directions, respectively. Figure 4(a) shows a photograph of the mouse tumor, and Fig. 4(b) shows the corresponding *in vivo* PA maximum amplitude projection (MAP) image prior to the injection of the PB NPs for the excitation wavelength of 532 nm. It is noted that PAI is used for tumor detection due to the presence of dense vasculatures at the tumor location when compared to that of normal tissues, and this enhances the PA image contrast^16,35^. Here the PA image contrast is defined as follows:

1 $$PA\,image\,contrast=\frac{P{A}_{contrastagents}-P{A}_{background}}{P{A}_{background}}$$

where *PA*_*contrast agents*_ denotes the PA signal amplitude of the contrast agents and *PA*_*background*_ denotes the PA signal amplitude of the background tissue. In the present study, the contrast agents can be endogenous (e.g., blood) or exogenous (e.g., PB NPs). As seen in the control PA MAP image in Fig. 4(b), the dense vasculatures in the tumor site are clearly observed with a high contrast (6.73 ± 2.30). Following the intratumoral injection of PB NPs into the tumor [Fig. 4(c)], a series of consecutive PA images were acquired, as shown in Fig. 4(d–f). In the PA MAP image acquired using the 532 nm wavelength immediately after injection [Fig. 4(d)], the dense blood vessels are still clearly observed, while the injected NPs are barely visible (7.66 ± 2.17) due to the low optical absorption coefficient of the PB NPs at the selected wavelength. Then, we switched the SMF by a PM-SMF to perform tunable-color PAI in the same region [Fig. 4(e)]. The full spectrum at the fiber output covers the spectral window of ~532–712 nm, as shown in Fig. 2(b). In this case, the PB NPs are clearly visible with a two times higher contrast (15.70 ± 2.54) because of the stronger optical absorption of PB NPs for red-shifted wavelengths. Finally, we used a 25 nm bandpass filter with a center wavelength of 700 nm (#86–646, Edmund Optics) that partially covers the peak absorption of the PB NPs [Fig. 2(c)]. The measured pulse energy at the desired band was 0.2 µJ. Figure 4(f) shows the bandpass-filtered PA image of the same area. In this case, the NPs are still visible with a low contrast (1.60 ± 0.25), while blood vessels are not observed due to the extremely low optical absorption of blood at the selected wavelength band. Additionally, the depth of focus of the laser beam is narrow (~55 µm) for the current bandpass wavelength, so that the pulse energy is not sufficient to generate PA signals from the deeply located NPs.

## Discussion

The current study demonstrates the feasibility of *in vivo* monitoring of PB NPs injected in a mouse tumor by using a newly developed OR-PAM system based on a tunable-color SRS light source. OR-PAM systems can be built to operate in the following two modes to achieve confocal alignment of the optical and acoustic beams: transmission mode and reflection mode. In the transmission mode^33,34^, the optical objective is opposite to the ultrasound transducer while the sample is at the middle. Although this configuration is relatively easy to implement, the application of the transmission-mode OR-PAM is considerably limited to only thin samples. Thus, it is not suitable for many *in vivo* applications and especially for the imaging of mouse tumors. In contrast, in the reflection-mode OR-PAM, both the objective and the transducer are typically located on top of the sample. Therefore, reflection-mode OR-PAM is not limited by the sample thickness. However, it involves more complicated implementation to achieve the confocal alignment of the optical and acoustic beams. Typically, a beam splitter that consists of an uncoated prism and a metal-coated prism, which is optically reflective albeit acoustically transparent, is used for acoustic-optical coaxial alignment^32^. Alternatively, a configuration with two prisms that sandwich a special silicone oil layer, which is optically transparent albeit acoustically reflective, is used^41,42^. The disadvantage of these configurations is that only a flat transducer glued to the prism can be used. An acoustic lens must be attached or ground at the bottom of the other prism to achieve ultrasonic focusing. The most significant disadvantage of these designs is that the acoustic impedance mismatch between the acoustic lens (glass: 13.1 MRayls) and load medium (water: 1.5 MRayls) causes an acoustic energy loss of up to 30%^43^. The acoustic energy loss inside the combiner also reduces the sensitivity of transmission-mode OR-PAM systems. In this study, we developed an OABC that allows the use of both flat and focused transducers to redirect the acoustic beam. Our system directly uses a conventional immersion focused transducer that was originally coated with a water impedance matching layer to minimize the acoustic energy loss without using any acoustic lenses.

SRS light sources for PAI have been studied for several years^31–34^. In the first report of an SRS light source, individual pulse energies of less than 90 nJ were obtained at 532 nm, 580 nm, and 600 nm for spectroscopic PAI in phantom studies^31^. With respect to higher pulse energies, an SRS laser source that uses a large-mode-area photonic crystal fiber was reported with a pulse energy of up to 530 nJ at wavelength of 589 nm^32^. However, an SRS laser source with a broader spectrum and high individual pulse energy is required for practical *in vivo* applications of PAI-guided cancer treatment. Hajireza *et al*.^34^ demonstrated that an SRS laser source can generate spectral peaks with wavelengths of up to 788 nm and with overall pulse energies of hundreds of nJ. However, previous studies have not exploited the potential of an SRS laser source for PAI monitoring of NP targets in a specific narrow wavelength region (i.e., around the absorption peak wavelength of PB NPs at 712 nm). It is also important to note that the key requirement for the light source for spectroscopic PAI is to deliver sufficiently high pulse energies, on the order of hundreds of nJ, at each individual specified wavelength region output. In the present work, a 700 nm filter with a bandwidth of 25 nm at full width at half maximum (FWHM) is used, with the pulse energy of this selected narrow wavelength region of more than 200 nJ at the fiber output, which is a sufficiently high pulse energy for *in vivo* PAI, as shown in Fig. 4(f).

To overcome the imaging depth and contrast limitations due to strong light scattering in biological tissue for functional and structural PAI, multiple wavelengths are used simultaneously to bring the total pulse energy up to 1.5 µJ and extend the depth of field to more than 200 µm through the focal length shift induced by wavelength turning. Herein, an achromatic doublet lens (OL2 in Fig. 1) was used to control chromatic aberration and achieve a nearly diffraction-limited spot. According to the lens raw data (AC254-050-A, Thorlabs), the focal length shifts by a distance of 160 µm when the wavelength changes from 532 nm to 700 nm. Furthermore, we theoretically determine the depth of focus (DOF) as ~42 µm and 55 µm at wavelengths of 532 nm and 712 nm, respectively, using the following expression:

2 $$DOF=\frac{8\lambda {F}^{2}}{\pi {D}^{2}}$$

where *λ* denotes the wavelength, *F* denotes the focal length (50 mm), and *D* denotes the collimated beam diameter at the back aperture of the lens (9 mm). Therefore, our imaging system provides better image quality [Fig. 4(e)] owing to (1) a slight increase in the depth of field for structural imaging due to a shift in the focal length and (2) an improved imaging contrast due to both the unscattered and scattered light in the biological tissue.

The theoretical laser spot size (2ω_0_) is calculated as follows:

3 $$2{\omega }_{0}=\frac{4\lambda F}{\pi D}$$

The lateral resolution of the OR-PAM system is estimated as equal to the laser spot size and corresponds to 3.8 µm and 5 µm for wavelengths of 532 nm and 712 nm, respectively. A trade-off exists between the penetration depth and lateral resolution in OR-PAM. An increase in the penetration reduces the lateral resolution. The axial resolution of our system is measured as 43.5 µm, extracted from time-resolved ultrasonic detection of a 6-µm carbon fiber at a wavelength of 532 nm (Fig. S1). The axial resolution depends mainly on the properties of the transducer (i.e., the center frequency and fractional acoustic bandwidth); it is considered constant when using multiple wavelengths. With all the wavelengths combined, the system exhibits a lateral resolution close to that of single-wavelength imaging^33^. However, the ratio of the main-lobe level to the side-lobe level is decreased, leading to a reduction in the SNR (14.14 dB for 532 nm vs. 12.23 dB for 700 ± 12.5 nm and 11.82 dB for all wavelengths). This trade-off is expected because the out-of-focus wavelengths will add some of the off-target signal to the in-focus signal due to both the presence of unscattered and scattered light in tissues. Blood oxygenation levels may also affect the main absorbing wavelength and thereby the depth-dependent resolution. One of the most suitable optical wavelengths for visualizing the superficial vasculatures is 532 nm while far-red and infrared light would be optimal for deep tissue imaging and strongly absorbed by PB NPs. In the red region, the optical absorption coefficients of blood with 90% and 10% oxygen saturation are less than 7.6 cm^−1^ (3.2% of that at 532 nm) and 30.6 cm^−1^ (13.8%), respectively. Therefore, in conjunction with PAI, the tunable-color SRS light source visualizes PB NPs and blood vessels as compared with the background tissue (Fig. S2). Additionally, our results agree well with the wavelength-dependent optical absorption coefficients of blood and PB NPs.

The numerical aperture of the used objective lens is 0.12 in water. Given the maximum pulse energy (for a single wavelength) of 2.26 µJ focused to a depth of 0.5 mm under the mouse skin surface, the laser surface fluence is estimated as 20 mJ/cm^2^, which corresponds to the American National Standard Institute (ANSI) safety limit for a wavelength under 700 nm^44^. The actual average pulse energy for the single wavelength of 532 nm and for the full spectrum are ~0.8 µJ and 1.5 µJ, respectively. The corresponding laser surface fluences are 7.07 mJ/cm^2^ and 13.26 mJ/cm^2^, which are still well below the ANSI limit.

In this work, we used a low dose of 2.5 mg/kg of bare intratumorally injected PB NPs, which were fabricated from low-cost chemical agents without surface engineering and coating. Thus, bare PB NPs might not exhibit optimal performance in biological systems for selective detection of cancer via intravenous injection. In a previous study^2^, PB NPs were carefully coated with polyethylene glycol and used as a multifunctional nano-platform for cancer diagnosis and therapy through *in vivo* magnetic resonance/PA tomography bimodal imaging and PPT, thereby revealing efficient passive tumor targeting of the polymer-coated PB NPs after intravenous injection without toxicity even at a high dose of 20 mg/kg, which is eight times higher than the dose used in our study. In a similar way, PB coated gold NPs for simultaneous photoacoustic/computed tomography bimodal imaging and photothermal ablation of cancer were performed *in vivo*^3^. These advantages provide PB NPs with immense potential as a new-generation far-red laser-driven PTT/PAI agent, alternative to traditional agents for cancer treatment and guidance. We note that PB is typically taken orally in doses (e.g., 9 g per day for at least 30 days for adults), which demonstrates its approved biosafety for the human body. It is also important to note that PB is approved by the FDA for clinical application; however, the approved is only for bare PB NPs but not for general NPs.

In conclusion, we found that the strong optical absorption of PB NPs around 700 nm matches the output spectrum of the SRS light source, which allows both deep tissue imaging and strong absorption by the NPs as compared with the background tissues. Moreover, the similarities between the fiber-based tunable-color SRS light source and fiber-based OR-PAM system facilitate the development of a hybrid technique based on these two modalities, with several advantages such as compact and simple system implementation and cost-effectiveness. Once PB NPs are injected into the tumor, OR-PAM imaging noninvasively monitors the presence of NPs and blood vessels in the tumor sites with micrometer resolution; in the present work, this was achieved for the first time with the use of an SRS light source. We expect that this method provides a new laser source for PAI-guided cancer treatment with a reasonable price and approachable solution. The results of this study suggest that PAI based on a tunable-color SRS light source is a useful tool to assist in cancer therapy.

## Methods

### Tunable-color OR-PAM system

A Q-switched diode pumped solid-state laser (SPOT-10-100-532, Elforlight) was employed as a pumping source producing 16-µJ pulses at a wavelength of 532 nm. The laser was operated at a repetition rate of 5 kHz with a pulse width of 1.8 ns. Initially, the laser pulses were passed through a variable beam splitter/attenuator in conjunction with a zero-order half-wave plate (VBA05-532, Thorlabs) prior to coupling into a 35-m PM-SMF (PM-S405-XP, Nufern). The output light of the PM-SMF was band-pass filtered, collimated by a microscope objective lens (RMS4X, Thorlabs), and refocused by using another achromatic objective lens (AC254-050-A, Thorlabs) to achieve near diffraction-limited optical focusing. The focused laser beam was then excited a sample to generate PA waves and was confocally aligned with a spherical focused transducer (center frequency: 10 MHz, Olympus NDT) through an OABC to maximize detection sensitivity. The OABC containing a 1-mm thick glass slab and an air–water corrective lens was sealed with a 10 µm plastic membrane (PMB) before it was fully filled with water. An imaging window was positioned at the bottom of the water dish and sealed with another PMB for optical and acoustic transmission. The two-axis linear stage in synchronization with the trigger from the laser provided volumetric imaging of the sample by applying raster scanning of the OABC. The generated PA signals were then detected by a single-element ultrasound transducer, amplified by using two serially connected amplifiers (ZFL-500LN, Mini-Circuits), and finally acquired by a high-speed digitizer (NI PXI-5122, National Instruments). The acquired data were converted to PA images via Hilbert transformation.

### Synthesis of PB NPs

In a typical procedure, FeCl_3_ (5.4 mg) was dissolved in deionized water (20 mL) under magnetic stirring at 60 °C. For this, K_4_[Fe(CN)_6_] aqueous solution (20 mL, 1 mmol/L) was slowly added in a dropwise manner at room temperature. The reaction mixture immediately turned bright blue and indicated the formation of PB NPs. The PB NPs were washed with deionized water and isolated by centrifugation at 14,000 rpm for 15 min. Finally, the precipitate was dried in an oven under vacuum.

### Cell culture

A human breast cancer cell line (MDA-MB-231) was cultured in standard culture media recommended by the Korean Cell Line Bank. MDA-MB-231 were cultured and maintained in Dulbecco’s Modified Eagle’s Medium that consisted of 10% fetal bovine serum supplemented with 100 U/mL penicillin and 100 µg/mL streptomycin. The cells were incubated at 37 °C in a humidified atmosphere containing 5% CO_2_.

### ***In vivo*** experiments

All animal experimental protocols were reviewed and approved by the Pukyong National University Animal Care and Use Committee. And all animal experiments were performed in accordance with the National Institutes of Health Guide for the Care and Use of Experimental Animals. A normal BALB/c nude mouse (Orient Bio Inc.) weighing ~20 g was used for the *in vivo* experiments. Specifically, MDA-MB-231 cells (4 × 10^6^) were subcutaneously injected into the flank region of the mouse. With respect to the *in vivo* PAI, the tumor-bearing mouse was intratumorally injected with 100 µL of 0.5 mg/mL PB NPs (i.e., dose = 2.5 mg/kg) when the tumor volume reached approximately 93 mm^3^ as follows:

4 $${\rm{Tumor}}\,{\rm{volume}}=\frac{({\rm{Tumor}}\,{\rm{length}})\times {({\rm{Tumor}}{\rm{width}})}^{2}}{2}$$

The mouse was initially anesthetized with 3% isoflurane vaporized by using the inhalation gas (flow rate is 1.0–1.5 L/min). During the *in vivo* imaging experiments, the mouse was kept under anesthesia with 1% isoflurane and was positioned below the water dish. A thin layer of ultrasound gel was applied between the PMB and the animal skin surface. An electrical heating pad was used underneath the body to maintain the body temperature.

## Electronic supplementary material

Supplementary Information

## References

[1] Center, M., Siegel, R. & Jemal, A. Global cancer facts & figures. *Atlanta: American Cancer Society*, 1–52 (2011).

[2] Cheng L (2014). PEGylated Prussian blue nanocubes as a theranostic agent for simultaneous cancer imaging and photothermal therapy. Biomaterials.

[3] Jing L (2014). Prussian blue coated gold nanoparticles for simultaneous photoacoustic/CT bimodal imaging and photothermal ablation of cancer. Biomaterials.

[4] Chen Y-S (2010). Enhanced thermal stability of silica-coated gold nanorods for photoacoustic imaging and image-guided therapy. Opt. Express.

[5] Shah J (2008). Photoacoustic imaging and temperature measurement for photothermal cancer therapy. J. Biomed. Opt..

[6] Mallidi S (2009). Multiwavelength photoacoustic imaging and plasmon resonance coupling of gold nanoparticles for selective detection of cancer. Nano Lett..

[7] Kim C (2010). *In vivo* molecular photoacoustic tomography of melanomas targeted by bioconjugated gold nanocages. ACS nano.

[8] Kim S, Chen Y-S, Luke GP, Emelianov SY (2011). *In vivo* three-dimensional spectroscopic photoacoustic imaging for monitoring nanoparticle delivery. Biomed. Opt. Express.

[9] Wang LV, Yao J (2016). A practical guide to photoacoustic tomography in the life sciences. Nat. Methods.

[10] Weber J, Beard PC, Bohndiek SE (2016). Contrast agents for molecular photoacoustic imaging. Nat. Methods.

[11] Liang X (2013). Prussian blue nanoparticles operate as a contrast agent for enhanced photoacoustic imaging. Chem. Commun..

[12] Wang LV, Hu S (2012). Photoacoustic tomography: *in vivo* imaging from organelles to organs. Science.

[13] Oraevsky A, Karabutov A (2003). Optoacoustic tomography. Biomedical photonics handbook.

[14] Wang, L. V. *Photoacoustic imaging and spectroscopy*. (CRC press 2009).

[15] Gao F, Feng X, Zheng Y (2016). Advanced photoacoustic and thermoacoustic sensing and imaging beyond pulsed absorption contrast. J. Opt..

[16] Ku G, Wang LV (2005). Deeply penetrating photoacoustic tomography in biological tissues enhanced with an optical contrast agent. Opt. Lett..

[17] Yao J (2016). Multiscale photoacoustic tomography using reversibly switchable bacterial phytochrome as a near-infrared photochromic probe. Nat. Methods.

[18] Manivasagan P (2016). Doxorubicin-loaded fucoidan capped gold nanoparticles for drug delivery and photoacoustic imaging. Int. J. Biol. Macromol..

[19] Manivasagan P (2016). Paclitaxel-loaded chitosan oligosaccharide-stabilized gold nanoparticles as novel agents for drug delivery and photoacoustic imaging of cancer cells. Int. J. Pharm..

[20] Bharathiraja S (2016). Cytotoxic Induction and Photoacoustic Imaging of Breast Cancer Cells Using Astaxanthin-Reduced Gold Nanoparticles. Nanomaterials.

[21] Oh Y, Jin J-O, Oh J (2017). Photothermal-triggered control of sub-cellular drug accumulation using doxorubicin-loaded single-walled carbon nanotubes for the effective killing of human breast cancer cells. Nanotechnology.

[22] Koo J (2012). *In vivo* non-ionizing photoacoustic mapping of sentinel lymph nodes and bladders with ICG-enhanced carbon nanotubes. Phys. Med. Biol..

[23] Bharathiraja, S. *et al*. Chlorin e6 conjugated copper sulfide nanoparticles for photodynamic combined photothermal therapy. *Photodiagnosis Photodyn. Ther*. 128–134 (2017). 10.1016/j.pdpdt.2017.04.005 28465165

[24] Manivasagan, P. *et al*. Multifunctional biocompatible chitosan-polypyrrole nanocomposites as novel agents for photoacoustic imaging-guided photothermal ablation of cancer. *Sci. Rep*. **7** (2017). 10.1038/srep43593 PMC5333628 28252638

[25] Shokouhimehr M (2010). Dual purpose Prussian blue nanoparticles for cellular imaging and drug delivery: a new generation of T 1-weighted MRI contrast and small molecule delivery agents. J. Mater. Chem..

[26] Dunbar K, Heintz RA (1997). Chemistry of transition metal cyanide compounds: modern perspectives. Prog. Inorg. Chem..

[27] Bui NQ (2016). *Ex vivo* detection of macrophages in atherosclerotic plaques using intravascular ultrasonic-photoacoustic imaging. Phys. Med. Biol..

[28] Shi W (2010). Optical resolution photoacoustic microscopy using novel high-repetition-rate passively Q-switched microchip and fiber lasers. J. Biomed. Opt..

[29] Yao J (2015). High-speed label-free functional photoacoustic microscopy of mouse brain in action. Nat. Methods.

[30] Gao, F. *et al*. Single laser pulse generates dual photoacoustic signals for differential contrast photoacoustic imaging. *Sci. Rep*. **7** (2017). 10.1038/s41598-017-00725-4 PMC5429673 28377616

[31] Koeplinger, D., Liu, M. & Buma, T. In *Ultrasonics Symposium (IUS), 2011 IEEE International*. 296–299 (IEEE 2011).

[32] Loya, A. K., Dumas, J. & Buma, T. In *Ultrasonics Symposium (IUS), 2012 IEEE International*. 1208–1211 (IEEE 2012).

[33] Hajireza P, Forbrich A, Zemp RJ (2013). Multifocus optical-resolution photoacoustic microscopy using stimulated Raman scattering and chromatic aberration. Opt. Lett..

[34] Hajireza P, Forbrich A, Zemp R (2014). *In-vivo* functional optical-resolution photoacoustic microscopy with stimulated Raman scattering fiber-laser source. Biomed. Opt. Express.

[35] Ho, C. J. H. *et al*. in *Frontiers in Biophotonics for Translational Medicine* 75–109 (Springer 2016).

[36] Agrawal, G. P. *Nonlinear fiber optics*. (Academic press 2007).

[37] Prahl, S. *Optical absorption of hemoglobin* http://omlc.ogi.edu/spectra/hemoglobin/ (1999).

[38] Kollias, N. & Gratzer, W. Tabulated molar extinction coefficient for hemoglobin in water. *Wellman Laboratories*, Harvard Medical School, Boston (1999).

[39] Hoffman HA (2014). Prussian blue nanoparticles for laser-induced photothermal therapy of tumors. RSC Adv..

[40] Hecht EOptics (2002). 4th. International edition.

[41] Maslov K, Zhang HF, Hu S, Wang LV (2008). Optical-resolution photoacoustic microscopy for *in vivo* imaging of single capillaries. Opt. Lett..

[42] Hu S, Maslov K, Wang LV (2011). Second-generation optical-resolution photoacoustic microscopy with improved sensitivity and speed. Opt. Lett..

[43] Yao J, Wang LV (2014). Sensitivity of photoacoustic microscopy. Photoacoustics.

[44] ANSftSUo L (2007). American national standard for the safe use of lasers. *American National Standards Institute*. ANSIZ136.

