# Supplementary material for: In vivo photoacoustic monitoring using 700-nm region Raman source for targeting Prussian blue nanoparticles in mouse tumor model
Source: Sci Rep. 2018 Jan 31;8:2000. doi: 10.1038/s41598-018-20139-0 (PMC5792574; doi:10.1038/s41598-018-20139-0)
Supplement: Supplementary file 1 — Supplementary Information [file 41598_2018_20139_MOESM1_ESM.pdf]

SUPPLEMENTARY INFORMATION

In vivo photoacoustic monitoring using 700-nm region Raman source for targeting Prussian blue nanoparticles in mouse tumor model

Nhat Quang Bui[1†], Soon-Woo Cho[2†], Madhappan Santha Moorthy[3], Sang Min Park[2], Zhonglie Piao[2,5], Seung Yun Nam[1,3,4], Hyun Wook Kang[1,3,4], Chang-Seok Kim[2], and Junghwan Oh[1,3,4*]*

[1]Interdisciplinary Program of Biomedical Mechanical & Electrical Engineering, Pukyong National University, Busan, 48513, Republic of Korea

[2]Department of Cogno-Mechatronics Engineering, Pusan National University, Busan, 46241, Republic of Korea

[3]Center for Marine-Integrated Biomedical Technology, Pukyong National University, Busan, 48513, Republic of Korea

[4]Department of Biomedical Engineering, Pukyong National University, Busan, 48513, Korea Republic of Korea

[5]Massachusetts General Hospital, Wellman Center for Photomedicine, Boston, 02114, United States of America

† These authors contributed equally to this work.

*** Corresponding author:**

Prof. Junghwan Oh[1.3,4*]
Email: jungoh@pknu.ac.kr (J. Oh). Tel: +82-51-629-5771; Fax: +82-51-629-5779.

Prof. Chang-Seok Kim[2*]
Email: ckim@pusan.ac.kr (C. Kim). Tel: +82-51-510-1365; Fax: +82-51-583-2063.

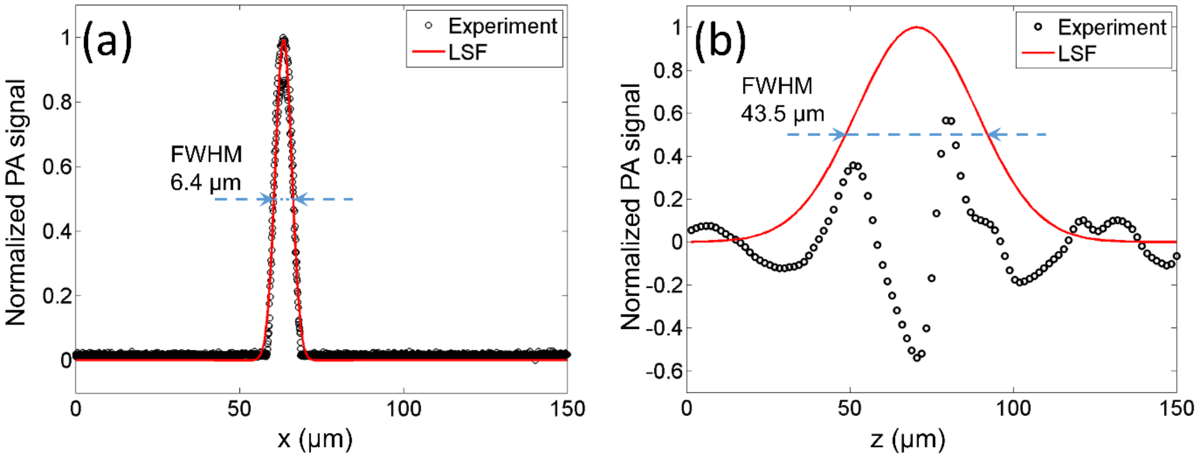

Supplementary Figure S1. (a) Lateral and (b) axial PA profiles of 6-μm-diameter carbon fiber using fiber-based OR-PAM system at 532 nm wavelength. FWHM: full width at half maximum; LSF: line spread function.

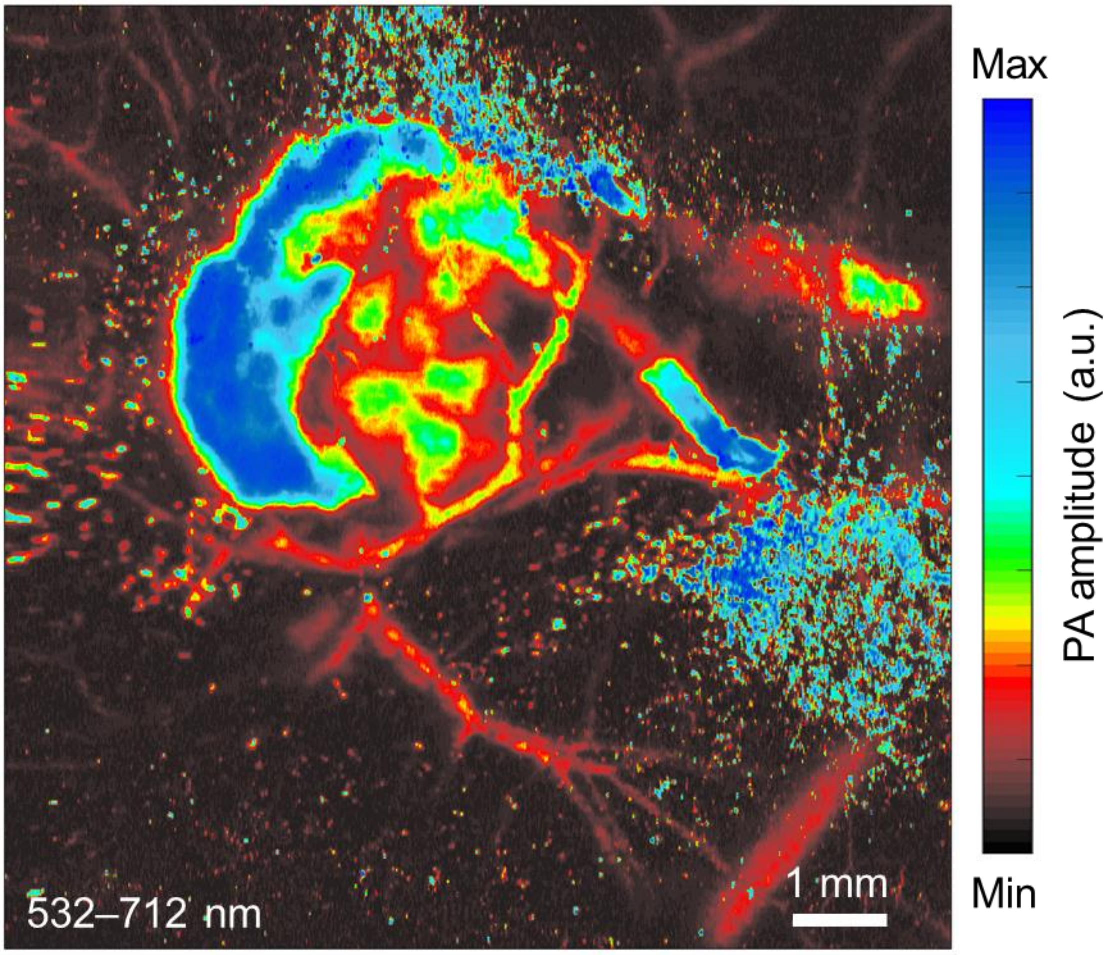

Supplementary Figure S2. *In vivo* PA amplitude-encoded monitoring of endogenous and exogenous contrast agents in mouse tumor after intratumoral injection of PB NPs under the excitation of tunable-color SRS light source.
